# REVIEW AND RECOMMENDATIONS FOR HEALTH INFORMATICS IN SUB-SAHARAN AFRICAN COUNTRIES: BETWEEN OPPORTUNITIES AND CHALLENGES

**DOI:** 10.18103/mra.v13i5.6554

**Published:** 2025-05-31

**Authors:** Olayemi Michael Lawanson, Daniel Berleant, Oluwatomiwa Ajiferuke

**Affiliations:** 1 Department of Information Science, University of Arkansas at Little Rock, USA; 2 Business Information and System Analytics Department, University of Arkansas at Little Rock, USA

**Keywords:** Healthcare, Informatics, Information quality, Health sector, Sub-Saharan Africa

## Abstract

**Background:**

There is a wide-reaching consensus that health should be a priority in national development plans. This is why the Sustainable Development Goal (SDG) 3 is aimed at securing good health for all. If the SDG 3 is to be a reality, there is a need to consider alternatives to the present traditional health system in sub-Saharan African countries. The implementation of health informatics, which is currently the focus of attention in the health sector, presents a huge opportunity with the potential to revolutionize the health systems of developing nations, particularly those in sub-Saharan Africa. In addition to reducing child mortality and infant death rates, providing high-quality healthcare in the region will help combat the challenges posed by preventable diseases and premature deaths that affect many sub-Saharan countries. Ensuring the quality of healthcare also depends on improving information quality, as accurate, timely, and reliable data are essential for effective decision-making and patient care. If the Sustainable Development Goals (SDG) is to be achieved, this issue must be addressed.

**Methods:**

This study reviews research on health information systems in sub-Saharan Africa. The search used medical databases (PubMed and Scopus) and found articles with keywords like "health," "information," "Africa," and "informatics." It also focused on both benefits and challenges of digital health systems in sub-Saharan Africa. The articles were categorized into health information’s potential benefits, its application, and barriers and opportunities to set up these systems. This review summarizes the current state of health technology in sub-Saharan Africa, identifies key trends and research gaps, and provides useful insights for health workers, governments, and NGOs.

**Results:**

Studies show that health informatics implementation in sub-Saharan Africa is still far behind where it should be. This analysis uncovers challenges in the deployment of health informatics into the health sector. It identifies infrastructure as well as other limitations and how they can be overcome to help enable the realization of quality healthcare in the region. This will improve the quality of healthcare information, helping to facilitate effective healthcare delivery. Documenting the insufficient current status of healthcare informatics in the health sectors of SSA countries’ health systems helps to highlight the potential contribution that informatics can make to health care in the region.

**Conclusion:**

The findings help provide a baseline of current knowledge to support efforts to implement health informatics in sub-Saharan Africa.

## 1. Introduction

### 1.1 MOTIVATION

The health of a nation’s people is a critical factor to its economic wellbeing. It is therefore important to protect the welfare of people through the prevention and control of health risks.^1^The Institute of Medicine^2^ in the United States has emphasized that information technology (IT) is indispensable for enhancing healthcare affordability, patient safety, and care quality while reducing disparities. Given these advantages, health informatics holds significant promise for developing countries, where systemic challenges—including economic constraints, political instability, and inadequate infrastructure—have long hindered healthcare delivery. Health informatics leverages information technology and computer science to transform healthcare by optimizing the storage, retrieval, and application of medical data.^3^ According to Yogesh & Karthikeyan,^4^ health informatics is a critical field that bridges healthcare and information technology, playing a vital role in modern healthcare systems. By leveraging technology to organize and analyze medical data, it enhances patient care, operational efficiency, and health outcomes. The COVID-19 pandemic highlighted the value of digital health solutions such as EHRs, telemedicine, and data analytics, which supported remote care delivery and minimized in-person contact.^5^These innovations empower healthcare professionals to make data- driven decisions, ultimately improving the quality of care.

There is a large body of literature relating social factors including poverty, race, ethnicity, marginalization, and physical environment to healthcare access and the spread of infectious diseases, such as influenza, malaria, tuberculosis and ebola.^6,7^ Several developing countries in Africa suffer from a shortage of well-trained healthcare providers and brain drain of health professionals. According to Fraser and McGrath,^8^ there are approximately ten doctors per 100,000 people, which varies from country to country. The healthcare professionals who serve the population in various Sub-Saharan African (SSA) nations are located far from them. There is a lack of modern x-ray, computer axial tomography (CAT), and magnetic resonance imaging (MRI) equipment and qualified radiologists. Physicians and other professional shortages are now affecting SSA. While promising pilot projects demonstrate health informatics^1^ transformative potential, widespread and sustainable implementation remains elusive due to persistent barriers. Compounding these issues, inadequate infrastructure, financial limitations, and workforce gaps further impede the adoption of health informatics solutions.^9,10^

The continent has fewer than 10 doctors per 100,000 people.^11^ Fourteen of its countries had few or no radiologists, which lowers the quality of the information available to diagnose and treat their citizens. People also lack access to up-to-date healthcare information, with internet and library facilities scarce in those areas. Consequently, the information quality required for a successful healthcare system is also lacking in most SSA thereby hindering and preventing the delivery of quality healthcare.^12,13,7,14^ Such studies suggest that disparities in access to healthcare and quality information affect the health outcomes of the SSA region.

The health care situation does not appear to be improving in most sub-Saharan African countries (SSA) because of the persistent economic development problems and increasing population of these nations.^1^ No doubt, healthcare in SSA is faced with several challenges hindering the delivery of efficient and effective healthcare. Most of the countries in this region are yet to embrace health informatics tools and can be best described as still advancing in terms of modernity in their health systems. Through a review of key challenges—including infrastructure deficits, workforce gaps, and funding limitations—alongside emerging opportunities in telemedicine, electronic health records (EHRs), and mobile health solutions, this paper seeks to provide critical insights for stakeholders to address systemic barriers and advance healthcare in rural communities.

### 1.2 HISTORY AND STATUS

Health informatics started as an innovative field of study in the 1950s-1960s but has only recently started receiving public attention as a significant constituent of the several aspects of healthcare. The field has also been called healthcare informatics, medical informatics, and biomedical informatics. It may be described as information processing for healthcare through the application of various information technologies.^15^ It is recognized as a multidisciplinary field that uses health information technologies to improve healthcare services via any combination of higher quality, higher efficiency and creation of new opportunities.^16^ O’Donoghue and Herbert^17^ argued that this multidisciplinary field deals with the resources, devices, and methods required to “optimize the acquisition, storage, retrieval, and use of information in health and biomedicine.” In settings with limited resources such as that of the sub-Saharan African countries, application of technology driven systems is critical for judicious use of scarce resources.^18^

### 1.3 POTENTIAL

Data collection is an important component of public health systems because decision makers, policy makers and health service providers need accurate and timely data to improve the quality of their services.^19^ The need for information quality is high as it is a necessary component for vital decision-making processes that impact national socioeconomic well-being. This includes analyzing and predicting health outcomes such as mortality and disease outbreaks.^20,21^ In Africa, the advent of e-health has offered an exciting opportunity to reduce and control healthcare inequalities; however, it is imperative to note that despite such technologies, much still needs to be done to address the health inequalities still hindering development in most developing countries.^22,23,24,25^

Advancement of Information and Communication Technologies (ICTs) and demand for health information to inform policies, research, resource allocation, monitoring and evaluation of healthcare programs has had significant impact within the health sector in most developing countries.^26,27^ Medical informatics, at the intersection of information science, computer science and health care, includes clinical guidelines, formal medical language, standards, and communication systems.^28,29^ Advancements in ICT and its integration into the healthcare domain during the last quarter of the 20th century has dramatically changed medical practice resulting in the reconstruction of the traditional boundaries of medicine and its organizational structures in the developed world.^30,31^ It has that potential for developing countries as well

## 2. Methods

The review leveraged PubMed and Scopus databases to identify relevant literature. The study selection process employed three inclusion criteria: (i) articles including in their title the keywords "health," "information," "systems," "Africa," "informatics," or "developing countries," (ii) articles that are written in English, an (iii) articles which deal with opportunities and challenges for health informatics in Sub-Saharan African. The retrieved articles were classified into four broad categories related to health informatics in sub-Saharan African countries:

- the potential of health informatics;
- applications of informatics in the health sector;
- challenges facing the deployment of health informatics; and
- innovative approaches to overcoming obstacles to deploying health informatics in sub-Saharan Africa.

The reviewed articles provide comprehensive evidence on the current state of health informatics in sub-Saharan Africa, identify key themes, highlight research gaps, and offer evidence-based insights for stakeholders including policymakers, NGOs, and healthcare providers.

## 3. The Potential of Health Informatics

There are increasing reports of disparities in the health standards between developed countries and developing countries in sub-Saharan African. These differences include differentials in healthcare delivery, and in the skills required to provide quality health services. This is clear in reports from reputable organizations such as the World Health Organization on basic health indicators such as government health expenditures, the life expectancy at birth, the infant mortality rate, average number of people per doctor or nursing professional, among others. These reveal that there exists a huge gap between the developed countries and sub-Saharan African countries. However, there is a growing consensus that the wide gap in healthcare spending can be narrowed if African countries prioritize the health sector to the same extent as other sectors tied to economic growth. Currently, the percentage of GNP allocated to health in developing nations remains significantly lower than that of industrialized countries. With 24% of the global disease burden but only 3% of the world’s health workforce, SSA faces critical gaps in service delivery, data management, and access to care. Digital health solutions, including electronic health records (EHRs), telemedicine, and AI-driven diagnostics, offer pathways to bridge these gaps. However, realizing this potential requires overcoming infrastructural, financial, and policy barriers.^32^

If SDG 3, aimed at securing good health and promoting well-being for all is to become a reality, alternatives to the traditional health system must be considered. The deployment of health informatics offers new opportunities with great potential to revolutionize the health sectors of developing countries. Access to the internet has dramatically improved in the last 10 years, and all countries in Africa now have direct access to the internet.^33^ This step will further improve availability and access to quality and affordable healthcare services in SSA. It is also essential for sub-Saharan African countries to adopt technological changes specific to improving the health sector.^34^

Information and communications technology (ICT) has had a massive effect on healthcare as it raises the level of quality of care, secures patients’ privacy, and lowers operational and administrative costs.^35,36^ While knowledge and services are important in healthcare, they need technology to be accurate, efficient, and reliable.^37,38^ As this transforms the structures and organization of health systems, health care professionals will need to be up to date on emerging technologies and the ever-expanding knowledgebase.^39^ Therefore, healthcare professionals are not only challenged with keeping track of this medical and biotechnological knowledge, but also with acquiring ICT skills and relevant competencies to access and use this knowledge and information.^40^

The introduction of the internet and access to medical knowledge has led patients to be more assertive and able to use digital information to challenge providers’ decisions and demand better care. Health informatics provides the opportunity for health care organizations to improve the quality of patient care and safety and has the potential to reduce costs and improve efficiency in the workplace.^41,42^ Health informatics has also helped to improve record keeping in the health sector. Some advantages electronic records have over paper records include access to medical records from remote locations, improved speed and ease of retrieval of records, avenues to flag abnormal results and the elimination of handwritten prescriptions, which reduce the occurrence of prescription errors.^42,43^ Other benefits are simultaneous access to patient records by multiple users and the ability to perform data queries to inform decision-making. These potential benefits have enabled their wide acceptability in industrialized nations.^44^ Health Informatics is considered beneficial in areas such as population health, individual care, health systems and medical technology.^45^ Other important areas include disease surveillance and control and prevention of epidemic outbreak, clinical services and operations, administrative operations and others. Therefore, training on how to effectively use health informatics to facilitate effective coordination is considered important for national growth and development. Information technology has a bright future for healthcare managers, practitioners and the industry in sub-Saharan Africa despite the challenges that remain.

## 4. Applications of Informatics in the Health Sector

Health informatics uses information and communication technology (ICT) to enhance the delivery of quality health care. It integrates devices, data collection, storage, data management, data utilization and retrieval in health and medicine. According to the World Health Organization (WHO), the use of informatics in health is not merely about technology,^46^ but a means to desired outcomes such as better treatment decisions, safer care, higher quality service, active response to health needs and others.

Examining the treatment of patients with multiple myeloma in Hohn, Kwara State, reveals the significance of health informatics. Advanced imaging tests like MRI and CT scans are necessary for diagnosis, monitoring, and treatment planning of multiple myeloma, a complex hematologic disease that affects plasma cells and frequently manifests as bone lesions and skeletal-related problems. These imaging technologies are commonly employed in the management of both orthopedic and oncologic cases in facilities such as the University of Ilorin Teaching Hospital (UITH) Orthopedic Department. However, tracking patient imaging histories, coordinating multidisciplinary care, and conducting longitudinal research are all made more difficult by the lack of a fully integrated health informatics system. Such disparities both a problem and an opportunity in the larger framework of Sub-Saharan Africa. The problem is the lack of adequate digital infrastructure and trained personnel, while the opportunity lies in leveraging health informatics to improve diagnostic efficiency, clinical decision-making, and patient outcomes. Examining the treatment of patients with multiple myeloma in Ilorín, Kwara State, reveals the significance of health informatics. Bone lesions and difficulties connected to the skeleton are common symptoms of multiple myeloma, a complicated hematologic malignancy that affects plasma cells. In addition to improving Multiple Myeloma care, strengthening health information systems in organizations like UITH through clinical decision support tools, imaging data integration and electronic medical records (EMRs) would offer a scalable paradigm for health informatics reform throughout the area.

Well-structured health information systems (HIS) can help by producing, analyzing, storing, and sharing reliable and accurate information for decision support at all levels of the healthcare system pyramid. Consequently, investing in the implementation of HIS has become an important challenge for health care systems in developing countries. Given the significant health challenges and the need to achieve the Sustainable Development Goals (SDGs), particularly the third goal focused on "Good Health and Well-being," a high-performing Health Information System (HIS) would be vital in improving the monitoring of key indicators.^47^

Although having an HIS is a necessity for modern health systems, many developing countries still lack a good HIS and consequently have difficulties in obtaining reliable and accurate health related information. Thus, they are unable to meet the needs of the population.

Health informatics helps to reduce medical errors and costs. However, this important change in health care requires the assistance of people knowledgeable enough to use technical ICT skills to deliver high-quality patient care.

For years, healthcare organizations have known of the advantages of shifting away from the fee-for-service model into providing value-based care and informatics can aid in these efforts to offer systematic, evidence-based treatment that avoids unnecessary or overly expensive procedures. Clinical decision support helps create knowledge and informational support that is strategically placed at vulnerable decision points, allowing staff to consider safety, options for care, and education prior to proceeding with care decisions. Leadership can be supported by data with potential to shape the policy for the organization. Understanding what is happening in a facility can help leadership to determine educational needs, improve the quality of programs, and enable administrative efficiency.

Meindl^48^ summarized the areas of application in health informatics to include the following:

- *Medical research* – health informatics is useful for simulating biological systems, modeling disease processes, physiological interactions, pharmacological relations and others, and in biomedical research particularly in collecting and analyzing research data.
- *Medical data collection* – health informatics is also useful in standardizing patient medical records, permitting data management including data storage and retrieval of past consultations, and in tracking health outcomes and responses to treatment.
- *Medical decision-making* – health informatics can contribute to decision analysis, including clinical judgements and problem-solving paradigms for providing advice under conditions of uncertainty.
- *Clinical laboratories* – health informatics can help mechanical and electronic equipment receive and transmit information with low response time, errors and costs.
- *Interpretation of data from diagnostic tests* – health informatics is useful in flagging abnormal results from a large volume of data e.g. in electrocardiography.
- *Monitoring critically ill patients* – health informatics helps provide rapid and precise information about patients needed for accurate decision-making, e.g. in intensive care units.
- *Aids for the handicapped* – specialized systems can increase the abilities of the handicapped by implementing microelectronics-powered assistive devices.
- *Computed tomography* – a radiology technology that relies on specialized algorithms to control expensive devices and convert results into a useful form.
- *Ultrasonic imaging* – to produce an image of soft tissues such as a fetus, heart or other muscle using ultrasonic signals.

Every year, for example, the World Health Organization (WHO) publishes figures on the world’s health situation to measure member countries’ health outcomes. Improvement is needed and HIS can help. Through HIS, patients can keep track of their health status, and doctors can check the records of patients at their fingertips. Patients receive care simultaneously from multiple professionals in one stay.

Health informatics facilitates coordination by enabling healthcare professionals to record, share, and disseminate patient care records.

This literature review aims to provide an overview of the factors and challenges that influence the optimal functioning of HIS in counties with limited resources.

## 5. Challenges Facing the Deployment of Health Informatics

The challenges in deploying information technology to the health sector include inadequate resources, low technical skills among personnel, and the failure of many health systems to integrate the technology into their organizational structures. It remains unclear what types of systems can be best applied and why. This question cannot be adequately answered solely by theoretical debate, as it requires real-life evidence. Studies that illustrate successes or failures and identify factors responsible for outcomes are invaluable as pointers to what types of systems can be most effective.

In reviewing the literature, we have found challenges that hinder successful deployment of health informatics into health systems of sub-Saharan African countries. International development agencies argue that ICT is necessary to accelerate African socioeconomic development and bridge the digital divide between Africa and the industrialized world, especially in the health sector, due to its efficiency and effectiveness.^49^ African countries do not invest enough to train workers and to develop infrastructure. Lack of adequate finance, mismanagement, and bad leadership has hindered improvement of literacy levels even after many years of independence.^50^ African countries need to train more informaticians to develop and build ICT infrastructure and help formulate ICT policies, which would improve information quality in support of more effective health care.^51^

It has emerged that governance, a crucial element for efficient health informatics, has not been sufficiently considered in most of the developing world. The weak involvement of all stakeholders, together with weak leadership and insufficient coordination of health informatics actors are among the main factors that have threatened the use of health informatics in the regions’ health sector.^52^ One other impediment is the lack of exposure to readily available healthcare information systems. This goes along with the fact that most of the population is still not educated and lack information on how they can access appropriate health IT services.^53^

Limited resources, including human resources, combined with population growth in the developing world, put pressure on health data workers to manage large volumes of data using paper-based methods. This also makes it difficult for health service managers to track health data from remote health facilities. This may lead to delays in generations of important reports that could help decision makers and policy makers in improving the health services.^1^ Lack of adequate information may have negative impact on the health system such as underreporting important data.^54^ Data collection units at the operational level need to be equipped with appropriate technologies that can lead to availability of adequate and timely information. Health data systems in remote areas of the developing world have been facing problems in the reliability of data which hampers the delivery of quality health services.^55^ Poor infrastructure also increases the cost to set up and maintain the systems. In particular, there have been reports of power outages and network breakdowns; also, the need for parallel data entry contributes to overload on the limited health staff. Electricity supplies are often sporadic and inconsistent, the variations being hazardous to computer equipment, which must be protected from power surges and failures by stabilizers, uninterruptible power supplies, or generators.^56,57^

Health informatics is not sufficiently considered in national health policies, making it difficult to ensure the sustainability of health informatics.^52^ Inadequate infrastructure is another critical challenge preventing the smooth deployment of technology into the health sector of SSA countries. The technologies include poor internet penetration, as well as poor access to electricity across the continent on which digital solutions can be built.^58,59^ Many other aspects of support infrastructure are also relevant. Training facilities are generally poor, requiring staff, software, literature, equipment and other facilities that are not easily available locally. The existence of a national scientific infrastructure could help direct research efforts towards developing suitably small, cheap, self-maintaining systems for such environments. Emerging threats like cybersecurity breaches and ransomware attacks further jeopardize system integrity, while ethical concerns persist about Al diagnostic tools trained on non-representative datasets that may produce biased results for African populations. These multifaceted challenges demand comprehensive solutions that address both the technical limitations and the deeper structural barriers impeding digital health transformation across the region.

The organization of a complex health delivery system involves coordination at various levels. It also requires coordination between sectors that complement community development to reduce duplication and conflict among the various programs. It therefore includes the management of education, budgets, supply services, and personnel. The most obvious current deficiency is in administration at the district or local level, where technical capability is low. This link in the delivery chain is vital to maintaining contact with communities and responding to their evolving needs.

## 6. Innovative Approaches to Overcoming Obstacles to Deploying Health Informatics in Sub-Saharan Africa

A key enabler for access and use of ICT in healthcare delivery is adequate capital. Yet, there are limited investments in ICTs for health in most African countries.^60^ There is the need for governments of countries within the SSA region to increase funding to the health sector. This could drive investment in infrastructure sectors such as power supply and internet penetration among several others. This will provide a foundation for health informatics system development. Infrastructural support is certainly a necessary catalyst for the successful integration of health informatics into SSA health systems. As reported in a recent study, adapting telemedicine strategies to local realities in rural Nigeria has shown promise in addressing digital health challenges.^61^

Human resource development through appropriate education and training is a key factor in introducing new methods of work for healthcare service providers and in empowering citizens to use ICT to obtain medical information. The availability of an appropriate mix of competencies and skills is central to the wider diffusion of e-health. To improve the quality of the information that medical doctor’s access, it is essential that the relevant ICT skills be included in their training, so that, by the time they leave school, they develop expertise in the use of such technologies to solve health problems. Therefore, to improve the quality of the healthcare sector, the curriculum and training methods for health workers need to be considered. Only a knowledgeable and trained professional can contribute meaningfully towards the procurement process of ICTs for healthcare, but this is still a need in several SSA nations, as many of the health care professionals in the region lack the knowledge required.^62^

Educational programs are transforming the sector from the classroom-oriented model to a more online learning platform.^63^ This is driven by innovative ideas about healthcare education and modalities, which are shifting towards distant learning, telehealth, multimedia education and more emphasis on continuous learning through conferences, courses, and workshops, which, for example, Nigerian healthcare professionals can leverage.^64^

There is increasing evidence of developing countries bypassing governance policies in their efforts to adopt digital technologies across various sectors, including healthcare.^65,66^ There are countries in Sub-Saharan Africa that do not have a national digital health policy or strategy guiding the implementation and monitoring of health issues. There is, therefore, the need to consider amending laws to cover areas not covered by the existing laws.^67^ This would drive the health sector towards adoption of health informatics. This is necessary because ineffective leadership and monitoring have led to the unsuccessful implementation of most government policy programs on healthcare. There must be political willingness on the part of the governments of countries within sub-Saharan Africa for change. In that vein, a national health information management strategy should be put in place to facilitate positive healthcare performance in sub-Saharan African countries. The implementation of a health information system should consider the process of data collection, analysis, processing, and transmission at all levels of the health pyramid. It should support decision-making in health planning, epidemiological monitoring, and evaluation of health actions. This requires that the development of HIS be supported by sufficient governance with policies integrating health informatics aspects, including harmonized indicators, forms (electronic or paper-based) for integrated data collection, and databases that are easy to use and accepted by users.^68^ Sub-Saharan Africa is pioneering innovative, context-specific health informatics solutions to overcome infrastructure and resource challenges by using low-cost wireless clinic connectivity, AI-optimized health worker deployment, sustainable mobile money funding model, gamified learning and community-controlled health data, demonstrating how locally-adapted, low-cost digital health innovations can effectively address healthcare challenges in resource-limited settings while serving as models for other developing regions.

## 7. Recommendations

From the foregoing, it is important for sub-Saharan African (SSA) countries to take steps to establish and adopt health informatics in the region. It also suggests the need for investments in infrastructures to drive the process, build human capacity in areas of monitoring, analysis, and policy design, and where necessary, create new institutions to give the issue priority while developing Africa-wide health informatics capabilities. There is a need for government and donor agencies such as the WHO and the United Nations to collaborate to help ensure the provision of ICTs to SSA countries’ healthcare facilities. With this, it will become possible to successfully integrate health informatics into the healthcare systems of sub-Saharan Africa.

## 8. Conclusion

The present gap showing a significant deficit in the quality of healthcare delivery and health outcomes in the sub-Saharan countries can be addressed. The integration of health informatics into the health sector of sub-Saharan African countries has remained a great challenge for health care systems in the region. Given the significance of health to a nation’s development and the need to achieve Sustainable Development Goal (SDG) 3, “Good Health and Well-Being,” the application of health informatics in improving information quality and other health care challenges has great potential to play a major role.

Despite the challenges facing the health sector in SSA nations, there are great opportunities to be leveraged. In health informatics, if the governments of the countries that make up sub-Saharan (SSA) invest sufficiently in closing the healthcare technology gap and harness the potential of health informatics, yet no transformative policy changes are made, but no transformative policy changes are made, the impact may remain limited. Health informatics systems are practicable in the sub-Saharan Africa just like in other parts of the world and this review shows that the current healthcare systems are not sufficient to reverse the health care deficits in this region. There is a need for pilot projects to try adopting new models driven by information systems in a controlled and experimental way in the field. In conclusion, based on the current literature, most countries in sub-Saharan Africa as well as government institutions appear to be slow in implementing health informatics and other appropriate ICTs which are required to improve healthcare in the continent.
